# Advanced Strategies for Tissue Engineering in Regenerative Medicine: A Biofabrication and Biopolymer Perspective

**DOI:** 10.3390/molecules26092518

**Published:** 2021-04-26

**Authors:** Courtney R. Lynch, Pierre P. D. Kondiah, Yahya E. Choonara

**Affiliations:** Wits Advanced Drug Delivery Platform Research Unit, Department of Pharmacy and Pharmacology, School of Therapeutic Sciences, Faculty of Health Sciences, University of the Witwatersrand, Johannesburg, 7 York Road, Parktown 2193, South Africa; 2195369@students.wits.ac.za (C.R.L.); pierre.kondiah@wits.ac.za (P.P.D.K.)

**Keywords:** tissue engineering, biomaterials, natural polymers, cell regeneration

## Abstract

Tissue engineering is known to encompass multiple aspects of science, medicine and engineering. The development of systems which are able to promote the growth of new cells and tissue components are vital in the treatment of severe tissue injury and damage. This can be done through a variety of different biofabrication strategies including the use of hydrogels, 3D bioprinted scaffolds and nanotechnology. The incorporation of stem cells into these systems and the advantage of this is also discussed. Biopolymers, those which have a natural original, have been particularly advantageous in tissue engineering systems as they are often found within the extracellular matrix of the human body. The utilization of biopolymers has become increasing popular as they are biocompatible, biodegradable and do not illicit an immune response when placed into the body. Tissue engineering systems for use with the eye are also discussed. This is of particular interest as the eye is known as an immune privileged site resulting in an extremely limited ability for natural cell regeneration.

## 1. Introduction

Tissue engineering is a field of medical research that allows for the rebuilding and repairing of damaged tissues within the body. It encompasses a number of different aspects of science, including cell biology, engineering and medicine to formulate systems which are able to aid in the growth of new cells and tissues [1]. These systems have been investigated for almost every tissue in the human body, ranging from bone tissues, to cartilage and various components of the eye. For example, polymeric scaffolds have been shown to be a viable alternative to conventional grafts in the field of bone tissue engineering. These constructs (both those containing cells and those which don’t contain cells) are able to facilitate the required processes in bone regeneration [2].

One of the primary treatments for the severe tissue damage has been the use of either autogenous or allogeneic grafts. However, this treatment has its disadvantages including sourcing of the grafts, adverse immune responses and high cost. This opens the door for systems which are able to mimic the extracellular matrix and encourage the regrowth of cells and do not have these drawbacks. This has led to the investigation of systems such as the hydrogels and scaffolds which will be discussed throughout this article [3].

Due to the fact that tissue engineering can be used for so many different tissue and cell types, there are a variety of techniques and processes which can be employed in formulating these systems. For example, the physical design of the system can include (or be a combination of) hydrogels and 3D or bio-printed scaffolds. These systems need to be able to mimic the environment of the natural tissues of the body. In addition to this, the biochemical function of the systems can entail the delivery of biological signaling molecules, the delivery of new cells to the tissue, or simply a scaffold upon to which the body can naturally produce new cells [4].

When it comes to the delivery of cells to a damaged site, the methods that are currently used can be divided into two; to culture cells in vitro before they are implanted into the body as artificial tissue or to insert a graft (either laden with cells or without). The insertion of cells is complicated and has a high infection risk. It is also very costly compared to the insertion of a scaffold [5].

The very crux of tissue engineering is to create an environment adequate for the proliferation of new cells. One of the best ways to do this is to recreate the natural environment in which the cells normally grow. The extracellular matrix is the noncellular component of tissues which is responsible for structural support as well as the transport of biochemical cues. It also contains a variety of growth factors and cytokines which play a great role in the proliferation of cells. Decellularized extracellular matrix refers to matrix which has undergone procedures in order to remove all cellular components. The incorporation of this biomaterial into tissue engineering systems provides an environment which can support cell growth and viability while not producing the immune response which is commonly seen with transplanted tissues [6].

There are many important factors that need to be considered when designing a system for tissue engineering purposes; one of the most important is that the system be biodegradable so that the growing tissue is able to replace it as it degrades. Other factors which need to be considered include the mechanical properties, morphology and porosity of the scaffold. The ideal properties of tissue engineering scaffolds vary depending on the site into which it is going to be placed. The scaffolds must be mechanically strong enough to withstand being handled by surgeons during implantation as well as to be able to support the growth of cells and the movement of tissues. The flexibility of the scaffold may differ slightly; scaffolds placed into a bone would require less flexibility than that placed into soft tissue [7].

Biomaterials and natural polymers are a favourable option in these systems as many of them are inherently biocompatible and biodegradable [8]. Polymeric systems are able to replicate the extracellular matrix and allow for cell proliferation. For example, hydrogels are able to be administered to a damaged cartilage site and, not only alleviate the pain temporarily but also encourage neocartilage regeneration [9].

Tissue engineering systems are used in almost every type of tissue in the human body. Throughout this review, studies will be discussed which highlight systems which are being developed to treat damage to, among others, bone, cartilage, neural, ocular and skin tissues. Of particular importance is the development of neurological systems. There are a number of conditions (ranging from physical trauma to degenerative diseases) which lead to a loss of function within the brain and nervous system. Currently, the primary treatment strategies are only able to treat the symptoms of these conditions or slow the degenerative process. Tissue regenerative strategies are able to offer patients the possibility of long-term solutions [10].

## 2. Biofabrication Strategies for Tissue Engineering

The science behind tissue engineering systems is multifaceted and often dependent on the requirements of the tissues into which the system is being placed. Methods that are used in tissue engineering systems can be divided into many different strategies. These include the injection of cells that have been obtained from the patient into the damaged site, the administration of biomolecules such as growth factors or lyophilized cell fractions which are able to send cues to the patient’s own cells or the use of various matrices. These matrices include hydrogels and 3D structures (referred to as scaffolds) which mimic the extracellular matrix and can be used in conjunction with cells and biomolecules [11].

The design of a system is largely dependent on the tissues into which is going to be placed. Table 1 highlights some of properties that need to be taken into consideration [12].

It is important to keep in mind that these systems can be used in conjunction with one other. For example, as is demonstrated in many of the below studies, hydrogels can be utilized as a scaffold on their own or they can be further developed in into a bio-ink.

### 2.1. Hydrogels

A hydrogel refers to a system which is made of a network of hydrophilic polymeric materials that are able to interact with water without dissolving. These systems are useful in many biomedical applications, including drug delivery and tissue regeneration, for a number of reasons; the primary one being that they are largely biocompatible [13]. Due to the high level of hydrophilicity of hydrogels, they are able to very closely mimic the structural properties of the extracellular matrix in such a way that they are able to create an ideal environment for new cell growth. These cells are then in turn able to secrete new extracellular matrix. In addition to this, injectable hydrogels are able to be administered to the damaged site with minimally invasive techniques [14].

Despite these benefits, hydrogels are known to have poor mechanical strength, which can sometimes pose a challenge to researchers when developing systems for tissue engineering purposes [13]. The stiffness of the hydrogel plays an important role in the way it interacts with the tissues surrounding it and can affect the cell proliferation, differentiation, and adhesion. It is vital that this factor is optimized in order for the system to be as effective as possible [15]. In addition to adjusting the stiffness and mechanical properties of the hydrogel, other parameters are able to be finetuned in order to optimize the system. These include the degradation rate and the release kinetics of the substances within the hydrogel. An example of a method to optimize a hydrogel system in terms of its mechanical strength is by incorporating a second polymer into the network. This may be as an independent network, known as an interpenetrating network, whereby two or more polymer networks are present but are not covalently bonded to each other [16].

An example of such a system was developed by Saravanan et al., using chitosan, glycerophosphate and graphine oxide. Although chitosan has been used in many hydrogel systems, the researchers noted significant improvement in the physico-chemical properties of the hydrogel with the addition of graphine oxide. The system was able to support and promote differentiation of mesenchymal stem cells, making it viable option for bone tissue engineering purposes [17].

Some hydrogel systems are able to be designed to react to certain stimuli within the body. One of the most common stimuli-responsive hydrogels is those which are temperature sensitive. These, known as a thermosensitive hydrogel, are systems which are a liquid solution at a low temperature and undergoes the gelation process as the temperature is increased. These are beneficial for medical applications as they are able to be formulated to gel at body temperature. This allows for a hydrogel to be injected into damaged tissue sites as a liquid affording the hydrogel the ability to diffuse effectively through the tissue and fit within irregular tissue defects before it becomes a gel. In addition to this, thermosensitive hydrogels are able facilitate desirable diffusion of precursor elements through the system [18].

Yin et al., developed a thermosensitive hydrogel which was formulated using pluronic (P123) modified with butyl diisocyanate (BDI) and collagen (BC hydrogel) and seeded with tendon stem/progenitor cells for the purpose of tendon tissue engineering. The hydrogel had a sol-gel transition at 25 °C. The addition of collagen to the BDI hydrogel greatly improved its ability to support cells (pure BDI hydrogels have been shown to not support cell proliferation in a number of different cell lines) [19].

One of the challenges that face patients with tissue damage is the invasive procedure that is needed in order to insert tissue engineering scaffolds. Hydrogels, specifically those which are able to form in situ, are able to be injected which is much less invasive and therefore less uncomfortable for the patient. Sargeant et al., developed such a system with poly (ethylene glycol) (PEG) and collagen. The collagen allows for improved cellular adhesion and enzymatic degradation, where the PEG allows for hydrolytic degradation. In addition to this, it was found that a number of the characteristics, such as the mechanical strength, swelling and degradation profiles, were tunable. This illustrates just how adjustable hydrogel systems are and highlights that they are able to be formulated according to the specific requirements of each tissue site [20].

### 2.2. 3D Scaffolds

A second option for designing a tissue engineering system is a 3D scaffold. These scaffolds are intended to replicate the extracellular matrix while also providing structural support and allowing for the proliferation of cells [21].

When designing a solid 3D system, there are a number of factors and functions which need to be taken into account. These include promoting sufficient cell adhesion and regeneration, encouraging the transportation of substances such as nutrients and regulatory factors which are vital for cell survival and differentiation, degradation at a rate which equal to the rate at which the new cells are grown and to accomplish these factors without causing an inflammatory response [22].

3D printing of scaffolds has become a popular method of developing these systems as the resulting scaffold can be designed with a high level of complexity [21]. In the process of 3D printing, a solution (known as an ink) which contains a combination of substances including polymer, biochemicals and/or living cells, is placed in a layer-by-layer style in order to build up the scaffold. This is a particularly complex technique used in tissue engineering as not only do the inks used have to be able to emulate the micro-architecture found in the extra-cellular matrix, but they also have to be able to adapt from a liquid (when it is loaded into the printer as an ink) to a solid scaffold once it has been printed [23].

3D printed scaffolds provide a surface for the proliferation and regeneration of cells. In order for this to happen, these scaffolds need to have a structure which is very porous, with pores of a correct size to allow for the cells to infiltrate it adequately. The materials used in 3D printed scaffolds have to satisfy a number of parameters, not only do they need be biocompatible and able to allow of cell proliferation, they also need to be able to prepared in such a way that 3D printing is possible. This makes this form of tissue engineering systems particularly challenging [24].

The variety of possibilities and ability to ‘customize’ 3D printed systems gives researchers so many options when developing a scaffold for a specific tissue site. These range from the individual characteristics of different biomaterials to the structural possibilities of the 3D printing process. In a study by Boga et al., a cylindrical scaffold was developed with tricalcium phosphate, a bioceramic, and alginic acid and functionalized with graphine oxide, intended for use as a bone tissue engineering system. The design of the scaffold was created using computer-assisted software, with two different types of layers so that each would structurally support the next. As each layer was printed the scaffold was rotated 45°. This allowed for the creation of an interconnected pore network, with pores distributed evenly throughout the scaffold, while simultaneously keeping the mechanical strength intact. The results showed that the inclusion of graphine oxide had a number of beneficial affects; improved swelling profiles, increased porosity and improved mechanical strength. The 3D structure provides a surface onto which cells can adhere and proliferate. Scaffolds such as the one shown in this study provide an avenue to greatly improve the technology currently used in bone tissue engineering [25].

It is possible for systems to combine the benefits of a hydrogel with a 3D printed design. This was illustrated by Kilic and Hasirci et al., whereby a 3D printed methacrylated gelatin hydrogel was loaded with stromal keratocyte cells and printed utilizing a pneumatic extrusion based bioprinter. The design of the scaffold was created to mimic the corneal stroma by layering parallel fibres with a 90° difference between the layers. Through the mechanical strength of the bioprinted scaffold, coupled with the high cell viability of the hydrogel, the system is a viable alternative to allogenic grafts [26].

### 2.3. 3D Bioprinting

3D bioprinting, which refers to the use of inks which contain cells, is a type of 3D printing which is of particular interest in terms of tissue engineering. There are a number of different techniques which can be employed in 3D bioprinting formulations. The first of which is inkjet-based printing. This technique places small droplets of the bioink onto a substrate, either in a continuous stream or on a “drop-on-demand” basis. The second technique is extrusion based, where the ink is loaded into a syringe and forced out of through a nozzle, creating a continuous filament without the creation of droplets. Extrusion based printing requires the ink to be highly viscous whilst still being able to flow out of the syringe without the presence of an elevated temperature. The third technique, known as laser-based or orifice-free printing, involves the use of a laser to guide the placement of the ink onto the substrate [27].

**Figure 1:**
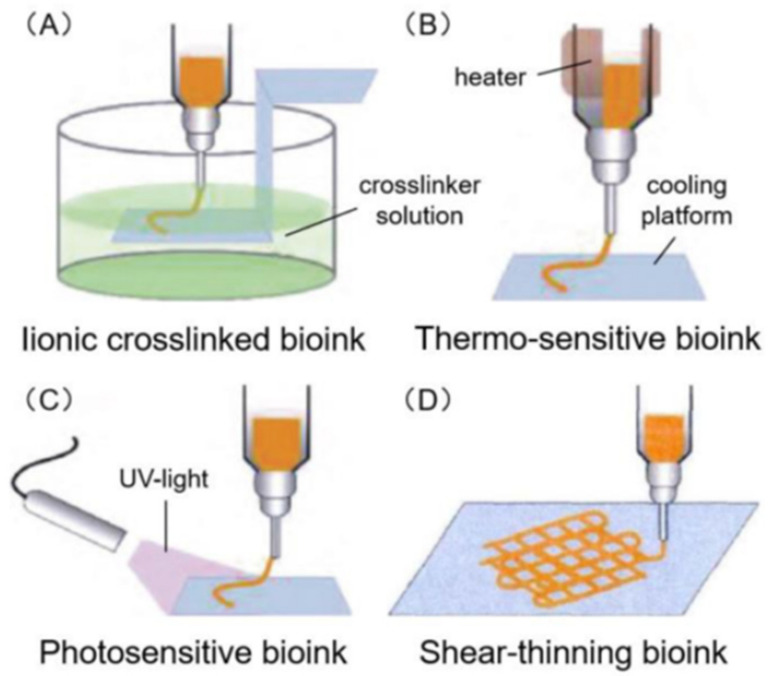
Illustrate above are the four common types of bioink. These undergo a transition from a liquid to a solid or gel as a response to either (**A**) an ionic crosslinking agent, (**B**) a change a temperature, (**C**) exposure to UV-light or (**D**) shear-thinning forces [28].

There are four of the common types of bioinks. These are primarily classified by the method through which they undergo the transition from a liquid to a solid or a gel. The first is through ionic crosslinking where the ink is printed directly into a crosslinking solution. The second is a bioink which is susceptible to changes in temperatures, it is a liquid at a higher temperature (within the syringe) and becomes a gel once it comes into contact with the platform which is a cooler temperature. Photosensitive bioinks react once they are exposed to UV-light. The last common bioink are those which undergo gelation due to the shear-thinning forces that it undergoes whilst printing. These bioinks are illustrated in the figure below (Figure 1) [28].

Lee et al., developed a 3D bioprinted system to mimic human skin whereby keratinocytes and fibroblasts are able to represent the epidermis and dermis. The system was comprised of a collagen hydrogel ink which was printed in a layer-by-layer design, alternating with the printing of cells. The final structure was made up of eight printed collagen layers. The fibroblast layers were printed after every two layers of collagen (three layers in total) and two layers of keratinocytes were printed on top of the last layer of collagen in order to replicate the cell density of the epidermis. The 3D printed system was tested against manually fabricated tissue samples with positive results. The printed system was able to maintain its shape, structure and physical dimension more effectively. This research showed that the 3D printing of skin tissues allows for improved control over the cell location as opposed to the traditional manual deposition method and is a feasible method for the creation and reconstruction of skin tissues [29].

### 2.4. Nano-Enabled Systems

A third avenue available to scientists when developing tissue engineering structures is through nanotechnology. Nanotechnology, which refers to structures and systems that fall within the nanometer scale, have gained much attention in recent years within the medical field. Researchers have found that the utilization of nanotechnology (including structures such as nanoparticles, nanofibers and nanowires) can greatly improve diagnostic capabilities, targeted drug delivery as well as tissue regeneration [30].

Nanotechnologies provide a particular benefit to tissue engineering systems (specifically bone tissue engineering) as they have a high surface to volume ratio allowing for improved tissue formation. However, bone tissue engineering system often require extra modification in order to be able to exhibit the necessary mechanical strength. This can be achieved using methods such as an apatite coating or the inclusion of titanium oxide [31,32]. Nanofiber scaffolds have adequate porosity throughout which allows for the infiltration of new cells while also possessing sufficient mechanical strength. The most promising and widely studied technique for producing nanofiber scaffolds is through electrospinning. This technique is able to produce nanofiber scaffolds from a variety of materials, including polymers and biomaterials. Researchers are also able to finetune certain characteristics of the resulting scaffold such as the fiber diameter and the surface morphology depending on the requirements of the tissue [33]. In addition to this, nanofibers are able to closely mimic the size of extracellular matrix proteins which are sized between 50 to 500 nanometers in diameter [34].

**Figure 2:**
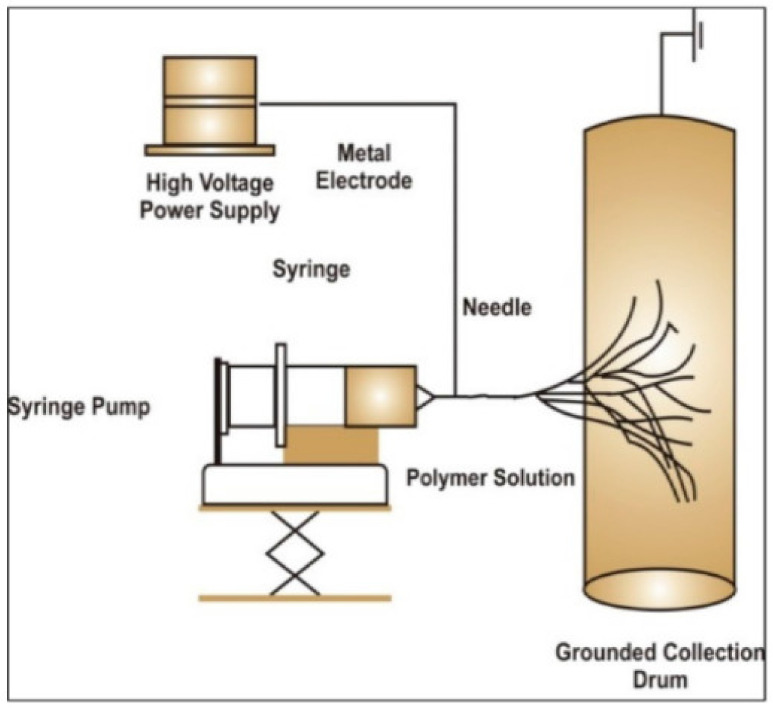
The setup of the process of electrospinning is depicted here. An electrical current is applied as the polymer solution exits the syringe at a steady rate set by the syringe pump. The resultant nanofibers are collected on the rotating collection drum [36].

There are three components which are required for the process of electrospinning, namely a polymer source, a voltage supply and a collector. The polymer solution is dispensed through a needle at a specific rate. When a high voltage is applied to the resulting droplet, the resulting electrical field overcomes the cohesive forces which are present in the solution, primarily in the form of surface tension. This then leads to a constant stream of polymer solution which is attracted to the collector. In the space between the needle and the collector, the solvent evaporates which causes the polymer to solidify in long fibers on the collector [35]. This process is depicted in the figure below (Figure 2).

In addition to the physiological benefits that nanofibrous scaffolds have with regards to cell regeneration, these systems are also able to deliver substances such as growth factors. These growth factors are vital for the stimulation of cell differentiation as well as extracellular matrix secretion. The delivery of growth factors cannot be done in a bolus fashion as they are readily broken down through enzymatic activity and are rapidly diffused from the site. Yang et al., developed a core-loaded nanofibrous system through emulsion electrospinning in order to deliver the growth factors on a continuous basis which allowed for improved wound healing [37].

A study by Sadeghi et al., was performed to investigate the utilization of a nanofibrous scaffold for the purpose of neural tissue engineering. The nanofibers were comprised of poly(ε-caprolactone) (PCL), chitosan and polypyrrole (PPy) and were formed through electrospinning. While the synthetic polymers, PCL and polypyrrole, offer characteristics such as PCLs ability to form fine fibers with the desired morphology and topography and PPys conductivity, the addition of chitosan was shown to be vital to improve the hydrophilicity (which is vital to the behaviour of the cells within the scaffold such as cell adhesion) and stability of the system as well as reduce the diameter of the fibers. This was illustrated through PC12 cell line and MTT assays. The samples which contained chitosan showed an 8.67-fold increase in the proliferation of the cells in comparison to that of pure PCL samples. These positive results illustrate not only the importance of the inclusion of biomaterials in these systems but also that nanofibrous scaffolds are a viable option for nerve tissue engineering [38]. 

The properties of nanofibers (such as the morphology) as well as the solution (such as the spinnability) are able to be optimized during formulation. This can be achieved by either adjusting the polymer solution concentrations or through the manufacturing process. This can be seen in a study by Wang et al., whereby nanofibers were formulated through electrospinning comprised on gelatin and pullulan (a microbial polysaccharide) for consideration as a tissue engineering scaffold [39]. In this study, the effect of a change in gelatin content on the properties of the solution and the resultant nanofibers was investigated. Pullulan was added to the solution as aqueous gelatin solution cannot be electrospun at room temperature. Solutions were formulated with a variety of concentrations (ranging from 20% to 25% *w*/*v*) and with different weight ratios of gelatin to pullunan (25/75, 33/67, 40/60, and 50/50). 

**Figure 3:**
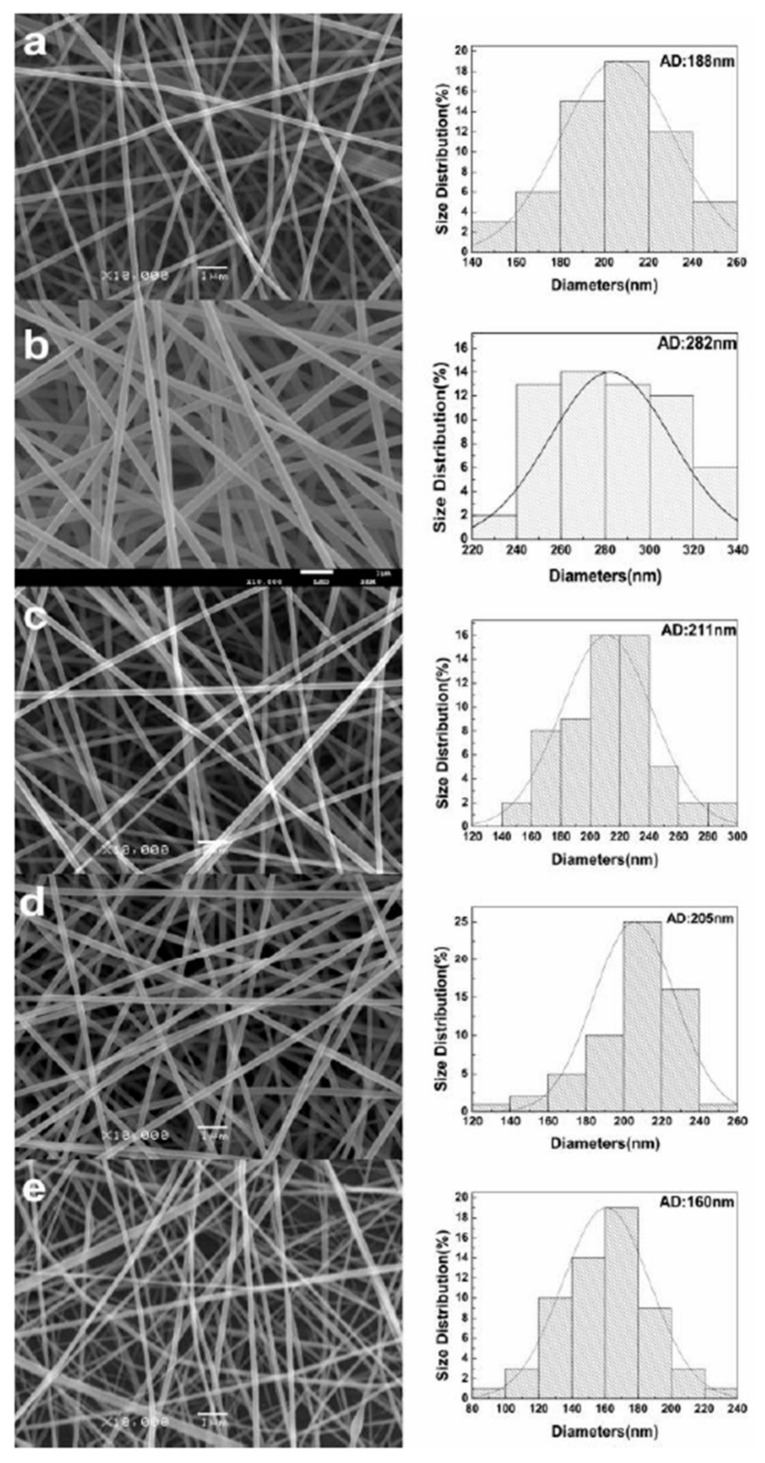
The SEM images of the resulting nanofibers as well as graphs of the average diameters are shown above. All the resultant nanofibers were bead-free. The concentrations (*w*/*v*) and mass ratios (chitosan/pullulan) of the solutions were as follows; (**a**) 20%, 25/75, (**b**) 25%, 25/75, (**c**) 25%, 33/67, (**d**) 25%, 40/60, (**e**) 25%, 50/50 [39].

The results showed that the viscosity of the solution increased as the weight ratio of gelatin increased. In addition to this it was noted that although the average diameter of the nanofibers increased as the solution concentration increased (from 188 nm in the 20% *w*/*v* sample to 282 nm in the 25% *w*/*v* sample) and the average diameter decreased as the gelatin content increased within the same solution concentrations. Through scanning electron microscopy (SEM) images seen in Figure 3, the changes in the diameter of the nanofibers can be seen as well as graphs showing the average diameter of each solution. This study highlights how adjustments in polymer solution can impact the optimization of nanofibers intended for use in tissue engineering [39].

Other nanotechnologies have also been used in tissue engineering formulations. One such technology is nanoparticles. In a study by Heo et al., a system was developed which illustrates how nanotechnology, hydrogel, and 3D printing can be used in conjunction with each other to produce a bone tissue regeneration system. A hydrogel was formulated using gelatin was used to reinforce a 3D printed microstructure composed of polylactic acid, a biodegradable thermoplastic, and produced using fused deposition modelling. The hydrogel was loaded with bioactive gold nanoparticles which reinforced the mechanical strength of the hydrogel. Through in vitro testing using human adipose-derived stem cells, it was shown that the cells remained viable within the hydrogel and were able to promote the proliferation of cells as well as the expression of osteogenic specific markers. This proves that this composite system may provide an avenue for improved bone tissue engineering [40]. 

## 3. The Utilization of Biopolymers in Tissue Regeneration Systems

Biopolymers have been extensively used in a number of medical and pharmaceutical applications, including drug delivery and tissue engineering. These materials originate from natural resources, as will be discussed below. These natural polymers can be used either on their own or in combination, with both other natural polymers and/or synthetic polymers. The addition of biopolymers to synthetic polymer systems increases the cell seeding efficiency as well as improves the hydrophobicity challenges seen with synthetic polymers [41].

In terms of tissue engineering, the selection of the materials used to formulate a system is a vital step. Not only does the architecture, including the topography, of the scaffold modify how the cells interact with the system, so does the materials which are chosen. This has been demonstrated in cartilage tissue engineering whereby different materials resulted in different levels of tissue formation [42]. Some of the biopolymers which are used in various medical fields are found naturally within the body. This contributes greatly to the biocompatibility of these polymers. Examples of polymers which occur within the human body include collagen and hyaluronic acid, both of which are found within the extracellular matrix of connective tissues [43].

### 3.1. Dextran

One of the sources used to obtain biopolymers is bacteria. Dextran, for example, is formulated from sucrose by lactic-acid bacterial species such as *Leuconostoc mesenteroides*. It has properties which lends it to use in pharmaceutical applications; it is highly water soluble, biocompatible, biodegradable and does not illicit an immune response when placed into the body. This makes dextran favourable when considering materials for tissue engineering scaffolds [44].

It was utilized in a system designed by Pan et al., in conjunction with gelatin to form a hydrogel with possible applications for cartilage tissue engineering [45]. The hydrogel was comprised of oxidized dextran and modified gelatin. The modifications were made to the biopolymers due to the fact that they do not possess the mechanical strength to withstand the force and load which is placed on cartilage joints. Researchers were also able to finetune the properties of the system to mimic the cartilage due to these modifications. The hydrogel was tested for cell viability using synovium-derived mesenchymal cells, a progenitor cell which are able to differentiate into chondrocytes. These cells were also loaded into hydrogels along with TGF-β3, a growth protein, and used in an animal testing model. The results showed that the dextran-gelatin hydrogel was able to support the growth of new cells as well as allow the mesenchymal cells to successfully differentiate [45].

### 3.2. Chitosan

Chitosan, a derivative of chitin, is one of the most abundantly available biomaterials for application in a variety of fields, including drug delivery and tissue engineering. It is able to lends itself to so many applications because it is biocompatible and biodegradable, offers mechanical strength to a formulation and is a cost-effective material [46]. This natural polymer is an amino polysaccharide which is derived from chitin by de-acetylation. Factors such as the degree of de-acetylation and the molecular weight have an impact on chitosan’s physicochemical properties. In addition to being biocompatible and biodegradable, it has antimicrobial, antioxidant and haemostatic properties [47]. 

Chitosan is favourable for application in tissue engineering as it provides a mechanical structure which closely mimics the extra-cellular matrix. Researchers are also able to modify both the pore size within the scaffold as well as the rate at which it degrades, allowing for adequate tissue integration within the scaffold. It is for these reasons that chitosan, both on its own and in conjunction with other biomaterials, has been formulated for the engineering of a variety of different tissues [48]. 

In a study performed by Dong et al., benefits of the addition of chitosan to a synthetic polymer system was shown [41]. In this study, a chitosan hydrogel was added to a 3D printed poly(ε-caprolactone) scaffold. The results, using rabbit bone mesenchymal stem cells as well as a growth factor, showed that the scaffold which contained the chitosan hydrogel had better cell retention and proliferation than the scaffolds without. It also had good mechanical strength, making the system a good candidate for bone tissue engineering [41].

**Figure 4:**
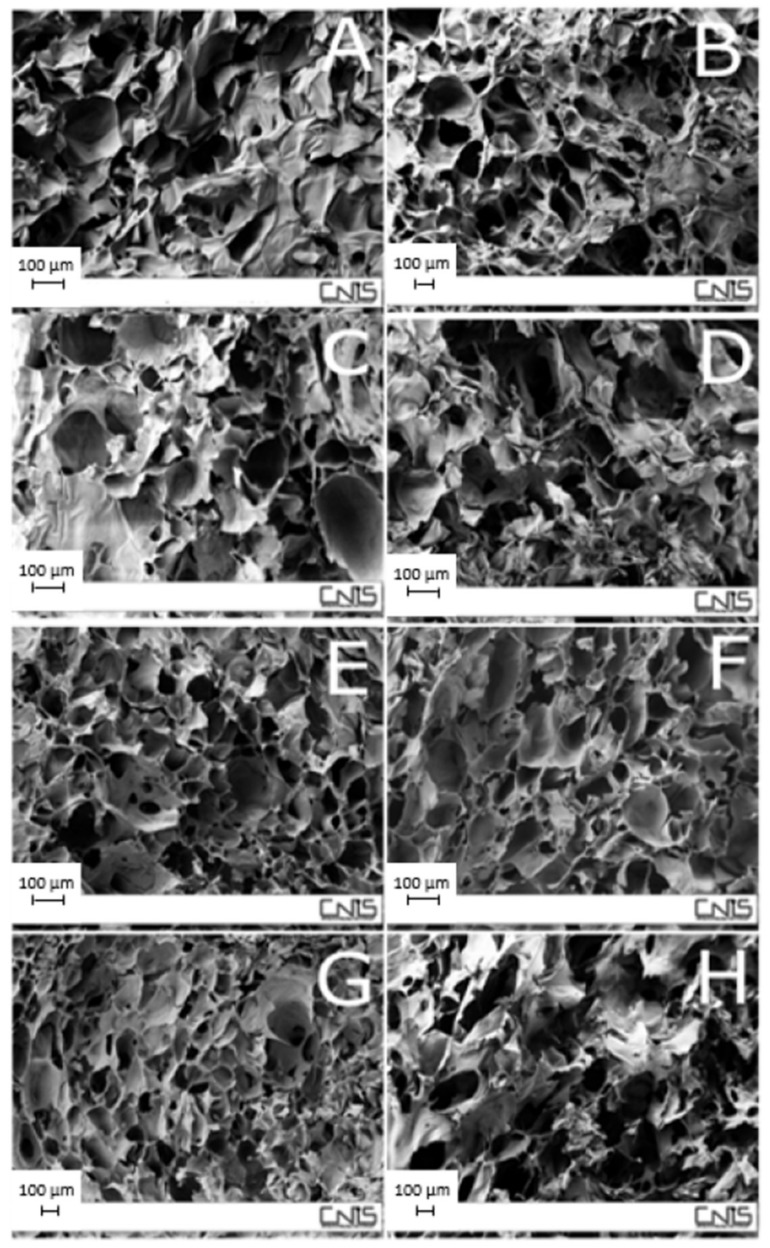
In order to view the porosity of the scaffolds formulated in this studied, SEM micrographs were taken. The concentrations and reaction times were as follows; (**A**) pure chitosan 1% *w*/*v*, (**B**) pure chitosan 2% *w*/*v*, (**C**) chitosan 1% *w*/*v*, TPP 1% *w*/*v*, 2 h, (**D**) chitosan 1% *w*/*v*, TPP 1% *w*/*v*, 8 h, (**E**) chitosan 1% *w*/*v*, TPP 2% *w*/*v*, 2 h, (**F**) chitosan 1% *w*/*v*, TPP 2% *w*/*v*, 8 h, (**G**) chitosan 2% *w*/*v*, TPP 2% *w*/*v*, 2 h and (**H**) chitosan 2% *w*/*v*, TPP 2% *w*/*v*, 8 h [49].

One of the most commonly used agents from the crosslinking of chitosan in tripolyphosphate polyanion (TPP). In a study by Silvestro et al., the optimal crosslinking conditions between chitosan and TPP, as well as the effect on the physico-chemical properties of the resulting scaffolds were investigated [49]. This is important as the porosity of a scaffold as well as the pore size play a vital role in cell adhesion and proliferation. The pores need to be within a suitable size range for successful interaction with cells. Scaffolds were prepared with different concentrations of chitosan (1 and 2% *w*/*v*) and TPP (1 and 2% *w*/*v*) as well as varying reaction times for crosslinking (2, 4, and 8 h) in order to optimize the formulation conditions. The figure below (Figure 4) shows the scanning electron microscopy (SEM micrographs of the various formulations. It was noted that there was more homogeneity in the pore size in the scaffolds comprised of 1% *w*/*v* chitosan and 2% *w*/*v* TPP (80–100 µm) compared to those comprised of 1% *w*/*v* chitosan and 1% TPP (50–20 µm). The results also showed that a higher TPP concentration and longer reaction times negatively influenced the resulting pore structure. This is like due to strong interactions between chitosan chains and the crosslinking agent. In addition to this, the scaffolds formulated with a chitosan concentration of 2% *w*/*v* had a more compact pore structure and low pore interconnectivity than the scaffolds formulated with a 1% *w*/*v*. Preliminary biocompatibility studies were completed on one of the scaffolds (chitosan 1% *w*/*v*, TPP 2% *w*/*v*, 8 h reaction) and the results showed good cell viability. This study illustrates how the aspects of a formulation can be modified to suit its intended design by adjusting parameters within the formulation processes [49]. 

### 3.3. Hyaluronic Acid 

Hyaluronic acid (HA), an anionic non-sulfated glycosaminoglycan also referred to as hyaluronan, is ideal for use in tissue engineering systems as it is naturally found within many connective tissues, specifically within the extracellular matrix. HA is also known to be found within synovial fluid, the vitreous of the eye, the nervous system and the skin. This highlights its biocompatibility and biodegradability. Other biological properties attributed to HA include anti-inflammation non-immunogenicity. The chemical structure of HA, including a variety of functional groups (carboxyl, hydroxyl and amide groups) allow for it to be easily modified to comply with the requirements of a specific tissue [50]. HA is able to interact with a number of different cells in order to affect aggregation, proliferation and migration [51]. Although HA has many beneficial properties, it does have some disadvantages such as its rapid degradation and poor mechanical strength. For this reason, many systems which incorporate HA often require chemical modifications or crosslinking in order to overcome these challenges [52]. This biopolymer is able to be formulated into a number of different systems including hydrogels and meshes [53]. 

In a study performed by Shu et al., a hydrogel film was formulated using hyaluronic acid and gelatin [54]. Both of the polymers were chemically modified to form thiolated derivatives (HA-DTPH and gelatiin-DTPH) before being crosslinked through a disulfide bond. The combination of these two polymers allowed the researchers to overcome challenges seen with each polymer. The enzymatic degradation of the gelatin-DTPH film was slowed by the addition of HA-DTPH, making them an option for long-term tissue engineering. In addition to this, when the gelatin-DTPH was added to the HA-DTPH films, the cell attachment to the hydrogel film surface was improved [54].

Another example of a hyaluronic and gelation combination system is that produced by Noh et al. In that study, a hydrogel was formulated with hyaluronic acid, hydroxyethyl acrylate (HEA) and gelation for use in bone tissue engineering, either as an injectable system, or as a bioink for a 3D bioprinted scaffold. The hydrogel was able to be loaded with bone cells in a viable manner, and successfully printed into a lattice form. This, coupled with the fact that the hydrogel was rheological stable and showed good biocompatibility, makes this system viable for bone tissue engineering [55]. 

### 3.4. Gelatin

Gelatin is a hydrolyzed collagen that has been receiving more attention recently in terms of tissue engineering systems. This is due to the fat that although it is biocompatible and biodegradable, in comparison to collagen it is more cost effective and has a lower antigenicity. As with many other polymers, gelatin is often chemically modified or combined with other natural or synthetic polymers in order to optimize its characteristics for the specific system [7].

The method by which gelatin is obtained from collagen; either through acid or base treatment which leads to the hydrolysis results in either type A or type B gelatin respectively. Each type of gelatin will have different characteristics such as gel strength, isoelectric point and charge. For example, the isoelectric point of type A gelatin is between 8 and 9, where it is between 4.8 and 5.4 for type B [56].

Gelatin is soluble in hot water, which gives it improved stability and therefore bioavailability when it is used within the body. Researchers are also able to alter the strength of a gelatin systems using various crosslinking methods, ranging from chemical (such as genipin and glutaraldehyde) to physical (such as UV radiation) [57].

Gelatin systems composed for nanofibers have been successfully investigated for tissue engineering purposes for a number of ocular tissues. This includes retinal tissues and corneal tissues. [56] An example of such a system was developed by Xiang et al. [58]. The system was designed to mimic the Bruch’s membrane, the layer of tissue which the retinal pigment epithelial cells (RPE) lie on within the eye. The damage or loss of these cells in diseases such as age-related macular degeneration (AMD) is one of the leading causes of blindness. This mimetic system, comprised of silk fibroin, polycaprolactone and gelatin, was shown to provide a long-term platform for the sustainable growth of RPE cells, without producing an inflammatory reaction and supporting the functionalization of the cells [59].

### 3.5. Alginate

Alginates, a group of natural polymers derived from algae and bacteria, are a favorable option for use in tissue engineering systems because they have properties which mimic the extracellular matrix within tissues [60]. Alginate presents with a highly beneficial biodegradability and biocompatibility profile as well as other useful properties such as mucoadhesion [61]. In addition to being negatively charged, alginates possess several beneficial properties; favorable solubility profiles, suitable porosity and shear-thinning capabilities. However, this biopolymer and its derivatives are also known to have poor mechanical strength and biological stability. These shortfalls can be overcome by crosslining (using either calcium, barium, magnesium or strontium ions) which increases alginates ability to form a gel or by oxidizing the alginate [62].

Sodium alginate, along with other natural polymers, have been formulated into a bioink designed to be used for 3D bioprinting in articular cartilage tissue engineering. The researchers found that the mechanical strength of the system was improved by the addition of collagen or agarose (when compared to the system with sodium alginate on its own) [63].

Alginate has often been used in combination with other biomaterials. An example of such a system was developed by Salehi et al., whereby alginate was combined with chitosan in a hydrogel loaded with olfactory ectomesenchymal stem cells for the promotion of peripheral nerve regeneration [64]. Once the hydrogel had been determined to be able to curate cell survival, it was tested using a rat model whereby sciatic nerve damage had been created in Wistar rats. The results of the MTT assay showed that the alginate/chitosan hydrogel was a more suitable substrate in comparison to the control group. During the in vivo model testing, common postoperative tests such as an electrophysiological assessment and gastrocnemius muscle wet weight-loss was conducted, and the results showed that the hydrogel was able to enhance the regeneration of the sciatic nerve [64].

## 4. Biocompatibility of Materials for Tissue Engineering Applications

One of the biggest factors that researchers need to take into account when designing tissue engineering systems is biocompatibility. Currently, even though the transplantation of tissues and organs from donors are widely used, patients are required to be treated with immunosuppressants for the rest of their lives to prevent the transplant from being rejected from the body. The development of systems which are biocompatible, biodegradable and are composed of the patients own cells would eliminate the need for immunosuppressant treatment [65].

There are a large number of tests which can be run in order to determine a systems biocompatibility. These tests can be in vitro, which refers to those conducted in a culture environment such as a test tube or petri dish, or in vivo, which refers to those conducted within a living model (usually an animal model). There are benefits to both in vitro and in vivo testing. In vitro studies are able to highlight the cytotoxicity and cell proliferation capabilities of a system and are able to give a preliminary view of the biocompatibility. In vivo studies are able to show how a system will react when placed into a body, for example inducing an inflammatory response. It is recommended that research groups include both in vitro and in vivo tests in their studies. This is because in vitro studies do not provide a full analysis as they are not able to illustrate how the system will interact with all the different cells and signaling factors that are found within the tissue [66].

Biocompatibility testing can be conducted on either on the components which make up the system or on the process by which the system is formulated. For example, the crosslinking agent that is used to formulate a hydrogel may negatively impact the overall biocompatibility of the system, even if it is comprised on known biocompatible polymers. This was shown in a study by Lai where he compared the biocompatibility of gelatin hydrogels which were prepared using glutaraldehyde (GTA) as a cross-linker to those prepared with 1-ethyl-3-(3-dimethyl aminopropyl)carbodiimide (EDC) [67]. The study included in vitro tests whereby rat iris pigment epithelial cell cultures were exposed to both hydrogels for 48 h. Following this cell proliferation assays, cell viability assays as well as pro-inflammatory gene expression tests were conducted. In vivo biocompatibility was also tested through an animal model. Samples of the hydrogels were inserted in the anterior chamber of the eyes of New Zealand white rabbits. Evaluations were then conducted at various time intervals over a 12-week period. The results showed that the hydrogels which were prepared using EDC were more biocompatible than those prepared using GTA. The EDC hydrogels expressed lower lactate dehydrogenase levels (this is released through cell lysis during proliferation assays) as well as lower interleukin-1β (IL-1β) and tumor necrosis factor-α (TNF-α) levels. In addition, the GTA hydrogels produced a significant inflammatory response in the animal model. This study illustrates well how biocompatibility studies are conducted [67]. 

## 5. Application of Tissue Engineering in Neurological Medicine

Tissue engineering can revolutionize the treatment of many diseases and disorder in the neurosciences. Several advances in this area have included the repair, replacement and regeneration of nerves, other soft tissues and organs such as the brain. The ultimate goal is to implant therapeutics to support regeneration of endogenous cells by creating a biomimetic environment. 

### 5.1. Neurology

Damage to the central nervous system can occur in a number of ways. These range from injury due to events such as car accidents and sports-related trauma, to neurodegenerative diseases such as Alzheimer’s disease and amyotrophic lateral sclerosis. The damage caused by such events leaves a lasting impact on patients as the central nervous systems has a very limited ability to self-regulate. This leaves a crucial area for investigation and development into possible tissue engineering solutions [68].

Tissue engineering systems are able to overcome the challenges that are encountered with autologous nerve grafts such as donor site morbidity and a limited supply of donor nerves. Neural tissue regeneration scaffolds are required to protect the regrowth of the nerve, particularly in the case of peripheral nervous system repair as it has a better ability to regenerate than the central nervous system, while also allowing for the delivery of biochemical signals. Biomaterials are able to provide the appropriate physicochemical properties (such as porosity), biomechanical properties (such as rigidity and flexibility) and biological properties (such as biocompatibility) [69].

In a study performed by Zarrintaj et al., a colloidal, drug-loaded hydrogel was developed with gelatin molecules and carboxyl capped aniline dimers [70]. The resulting hydrogel was electroactive, allowing for the close mimicking of the native environment and, in terms of neural tissue engineering, improve the response of the neural interface. The colloidal hydrogel was shown to have an optimal range for efficient cell proliferation and differentiation (10^−7^–10^−3^ S/cm). The electrical conductivity was also able to impact the drug release profile. The hydrogel was able to support optimal cell viability as well. Researchers noted that the characteristics (such as electrical conductivity and drug release profile) of the hydrogel were tunable, allowing for future investigations of the systems to adjust the resulting hydrogel to fit the intended characteristics. This system would be able to serve as a robust platform for a variety of cells and tissue regeneration, in particular neural systems [70].

The study above discussed how hydrogels may be utilized in neural tissue engineering. This is not the only system available to researchers. Saadatkish et al., illustrated how nanofibers have the potential to act as central nervous system (CNS) tissue engineering scaffold. The nanofibrous scaffold was developed using polycaprolactone, gelatin and fibrinogen. The synthetic polymer (polycaprolactone) provided the scaffold with mechanical strength while the natural biomaterials (gelatin and fibrinogen) provided improved hydrophilicity and cell proliferation. More particularly, it was noted that an increase in fibrinogen concentration allowed for higher proliferation of adipose-derived stem cells. However, too high a concentration led to weakened tensile strength, though not enough to impact the mechanical properties which are required for CNS tissue engineering. By increasing the hydrophilicity of the system, the biocompatibility of the scaffold was also improved. It was shown that the nanofibrous scaffold provide a suitable candidate for CNS tissue engineering applications going forward [71].

### 5.2. Otolaryngology

The field of head and neck surgery, including the repair of nasal, cochlear, laryngeal and tracheal tissue, has benefitted greatly from the advancement of local flap and donor tissue transplant technology. However, there are still challenges in this regard such as poor donor to recipient tissue matching, lack of donor tissue and transplant rejection. Tissue engineered systems are able to overcome these as they are able to be formulated so as to match the patient’s tissue and thereby prevent rejection [72].

An example of a system used in otolaryngology was developed and characterized by Ravanbakhsh et al. [73]. Carbon nanotubes were dispersed through a glycol chitosan matrix to form a composite hydrogel. The hydrogel was investigated as a treatment option following damage to the vocal folds which are responsible for producing the voice. Current treatment options for this condition includes materials such as collagen, hyaluronic acid and calcium hydroxyapatite which are injected as a synthetic extracellular matrix. However, these are associated with adverse reactions such as granuloma formation, over-stiffening of the material, chronic inflammation as well as low cell migration rates. The systems developed by researchers showed that the inclusion of carbon nanotubes into the hydrogel matrix allowed for a sizeable increase in pore size which is believed to be favorable to cell migration. The rheology, gelation time and cell viability of human vocal fold fibroblast cells also showed favorable results, which highlights the viability of this system as a synthetic scaffold for vocal folds [73].

In rhinology, tissue engineering can have multiple applications. These include reconstructive rhinoplasty, nasal septal repair and craniofacial reconstruction. The primary focus of these procedures is on the reconstruction of the cartilage [74]. The current primary source of cartilage is autologous grafts. However, this inevitably involves a need for additional surgical procedures in order to harvest the tissue (typically taken from the outer ear or rib. Synthetic implants, such as though comprised of silicone or polyethylene, are also used but these are known to have poor integration within the native tissue and are occasionally unnatural in appearance. In a study by Mendelson et al., a bioactive bilayered scaffold was developed comprising of a poly (lactic-co-glycolic acid) (PLGA) base topped with gelatin microspheres within alginate [75]. This system was developed as an alternative to current rhinoplasty procedures. The microspheres contained cytokines (TGFβ3) which were released over an extended period of time and promoted the growth of cartilage-like tissue. This system is an example of a cell homing system; the cytokine response recruits cells into the scaffold from the surrounding tissue which then differentiate accordingly [75].

The field of otolaryngology is vast and encompasses a number of tissue types within the head and neck. Tissue regeneration systems are in development for almost all of them. This includes the trachea. In a study by Jang et al., an electrospun polycaprolactone and collagen nanofiber scaffold was developed. In order to encourage cell growth once the scaffold was implanted, umbilical cord serum (which contains a number of growth factors and serum antiproteases) was introduced. The results showed that the scaffolds treated with umbilical cord serum had significantly higher cell viability than those which were not treated. The scaffolds were successfully able to facilitate both cell attachment and proliferation after seven days of cell culture. In vivo testing showed that the artificial trachea did not cause an inflammatory response. The system also showed complete regeneration of the tracheal wall. These results show that biomaterial nanofiber scaffolds are a viable option, not only for tracheal reconstruction but also enhanced cartilage and epithelial regeneration [76].

### 5.3. Ophthalmology

There are a wide variety of conditions and diseases which can lead to the damage of ophthalmic tissues. These include glaucoma, diabetic retinopathy, trauma or injury and age-related macular degeneration to name but a few. The damage that is caused to the eye is often irreversible and any vision that is lost cannot be recovered. Although much research has been done into the treatment of these conditions, many of the treatment options have risks associated with them such as redness of the eye and increased intraocular pressure. For many years, the primary treatment for many diseased or damaged tissues within the eye, for example the cornea, has been tissue transplants. However, this treatment method is heavily reliant on the availability of donor tissue, which is not always readily available and there is a history of rejection of allogenic grafts [77]. 

The eye is known as an immune privileged site. This means that the eye does not launch an immune response (for example against tissue grafts) in the way that some other organs do. Although this can be useful when it comes to introducing new substances to the eye, it also means that they eye is not able to regenerate tissues as effectively. This is what has led to the increased research that is being done into ophthalmic tissue regeneration technologies [78,79]. 

Corneal disease is the second leading cause of blinding, following behind cataracts, and while corneal transplant is the primary treatment option for many of these cases, a shortage in donations leads to many patients not being able to receive this life-altering surgery [80]. In a study performed over a 15-year period (2002–2016) in South Africa, it was shown that there was a progressive decline in the number of corneal donors from year to year [81]. This highlights the importance of developing systems which are able to replace or at least, alleviate, the need for human corneal donors. 

When considering tissue engineering intended for use in replacing a human cornea the two main factors which need to be optimized: tissue strength and transparency. It is these factors which make corneal tissue engineering the challenge that it is today [80]. 

The use of tissue engineering scaffolds for corneal tissues was demonstrated by Hsiue et al., whereby human corneal endothelial cells (HCEC) were transplanted into a rabbit cornea in the form of a cell sheet. The cell-laden disc was developed using poly(N-isopropylacrylamide) (PNIPAAm) and gelatin. Once the disc had been inserted, results showed that the gelatin disc was biodegraded and the cell sheet was integrated into the cornea successfully. This shows that the administration of cell-laden scaffolds is a viable option for the treatment of loss of corneal cell loss [82].

A further example, this time where mesenchymal stem cells were utilized, was developed by Goodarzi et al. The hydrogel system was composed of type-1 collagen and gelatin and was designed as an artificial corneal substitute. Collagen was chosen as a biopolymeric ingredient because it is a major component of the extracellular matrix and thus has exceptional biocompatibility capabilities and is able to promote cell regeneration. However, due to the high water content of pure collagen hydrogels, they have been shown to have poor mechanical strength and rapid degradability. These factors can be overcome either through the preparation of the hydrogel (for example using different crosslinking methods) or by the addition of another polymer. The addition of gelatin, a biopolymer with its transparency, biocompatibility and cell attachment capabilities, as well the implementation of zero-length EDC/NHS crosslinkers results in an adequate formulation. The resulting formulation was shown to be biocompatible, had positive mechanical strength and, with its porous structure, allowed for cell attachment and infiltration [83]. 

Retinal pigment epithelial (RPE) cells are found between the choroid and the neural retina within the eye. This highly specialized tissue is responsible for a number of functions including the transportation of substances such as nutrients and waste products to and from the photoreceptor cells. RPEs can be damaged by diseases such as dry age-related macular degeneration (AMD) which then leads to loss of vision as the RPE cells are not able to adequately support the photoreceptor cells. Currently, the transplantation of either donor or allogeneic RPE cells has been explored as a treatment for dry AMD. Although there have been positive results shown, there are issues regarding the positioning of the administered cells. It has been hypothesized that these issues may be overcome by administering cells through hydrogels or carrier systems in order to prevent the death of cells by reflux [84].

**Figure 5:**
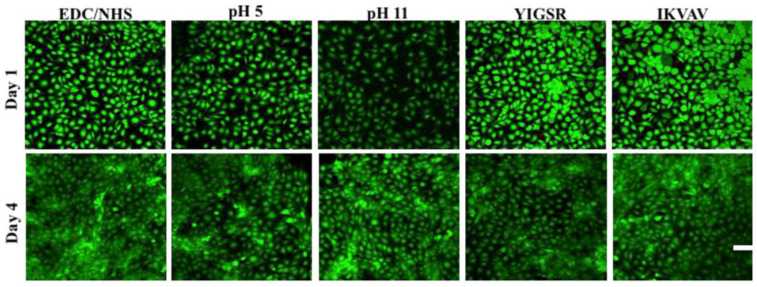
Confocal laser scanning microscope images were taken of each of the hydrogels at day 1 and day 4 with live/dead staining. The green fluorescence shows the live human corneal epithelial cells. (scale bar = 100 µm) [85].

In a study performed by Koh et al., a corneal substitute comprised of collagen in the form of a hydrogel was investigated. In addition to this, different crosslinking agents were utilized and the resultant hydrogels tested, as well as the effect on the addition of neurite promoting laminin epitope (IKVAV) and an adhesion peptide (YIGSR). The crosslinking agent in the first hydrogel was l,4-butanediol diglycidyl ether (BDDGE) and it was formulated at pH 11. In order to compare this with conventional methods, 1-Ethyl-3-(3-dimethyl aminopropyl) carbodiimide (EDC)/N-hydroxysuccinimide (NHS) which are commonly used crosslinking agents were also used in conjunction with BDDGE). Both crosslinking methods have previously been shown to improve mechanical properties of the collagen hydrogels such as elasticity and tensile strength. In this study the biocompatibility of the hydrogels was tested using human corneal epithelial cells. Confocal laser scanning microscopes were taken of the systems after live/dead staining (Figure 5). The results showed that the BDDGE hydrogels were non-cytotoxic with negligible dead cells indicating that it is biocompatible and comparable to EDS/NHS crosslinked hydrogels. In those with IKVAV and YIGSR there was increased cell proliferation but the elasticity of the hydrogels was negatively impacted. This study shows that BDDGE is a viable crosslinking agent from collagen hydrogels with a long gelation time that could allow for the encapsulation of drugs within the matrix as well which would further enhance the hydrogels for corneal substitution [85]. 

## 6. Strategies to Enhance the Bioactivity of Tissue Engineering Scaffolds

It has been mentioned that the incorporation of cells into a scaffold can be useful in the formulation of an engineered tissue. Primarily, cells from the patient have been used in this regard. However, researchers are now investigating the inclusion of stem cells into scaffolds. This is to avoid the limitations seen with the use of primary cells such as their potential to be diseased. Some stem cells that have been looked at are embryonic, bone marrow mesenchymal and umbilical cord-delivered mesenchymal stem cells [42].

Embryonic stem cells (ESCs) have benefits such as the ability to differentiate into multiple cell types and can also self-renew. However, there are ethical constraints which need to be taken into account when using these cells. Mesenchymal stem cells (MSCs) harvested from the umbilical cord blood have been shown to be a more viable option than ESCs as these have produced teratomas in the past. MSCs are also easier to obtain as there are cord blood banks available [86].

Adult MSCs are those which are harvested from bone marrow. Not only have they been investigated for tissue engineering purposes in a variety of tissues including cartilage, bone and muscle, they have also been shown to secret bioactive molecules which are able to encourage a self-regulated regenerative environment [87]. This means that not only are stems cells able to not only directly differentiate and regenerate new tissues, they are also able to regulate the immune response within the microenvironment of the damaged tissue and improve the regeneration of the tissues. The process by which MSCs are able to regulate the immune response is complex and involves the cell to cell contact between MSCs and cells which exhibit immunosuppressive properties [88].

While stem cells are able to provide an option for the development of new tissues, the transplantation of these cells on their own have shown poor viability and lowered regenerative capabilities. This opens the door for the further development and research into the tissue engineering scaffolds and systems which have been discussed in this article [89].

The incorporation of MSCs in a tissue engineering system was illustrated by Weinstein-Oppenheimer et al. The system, designed as a wound dressing system was formulated using gelatin, chitosan and hyaluronic acid to form a hydrogel. The resulting hydrogel had a porous scaffold which encourages the regeneration of cells. The system was tested both using an in vitro model using human cells as well as an in vivo model using rabbits. It was also applied to a single human case using autologous MSCs. Throughout the in vitro, in vivo testing and the human case, the system was shown to be biocompatible, had biodegradability capabilities (ranging between 1 to 2 weeks in the rabbit model and partial biodegradation after one week in the human model), good cell viability (99.5% after one week during in vitro testing) and an absence of rejection evidence in both the rabbit and human model [90].

In addition to the incorporation of stem cells, there are alternative methods which can increase the bioactivity of a scaffold. One such strategy is the utilization of materials which are better able to interact, or in some cases, bind with the tissue. This allows for improved cell growth and more stable anchoring of the scaffold to the tissue into which it is placed. This is of particular importance in bone tissue engineering systems. These materials are able to exhibit conductive properties and transmit biological cues, including growth factors [91].

It is also possible to improve the bioactivity of a scaffold by altering the biomaterials to mimic the extracellular matrix (ECM) more similarly. This can be done by modifying polymers with ECM-derived bioactive molecules, such as signal molecules. Proteolytic degradation and cell adhesion could also be used to improve the bioactivity of a material before it is incorporated into a tissue engineering system [92].

The choice of the structure which is being used to formulate a tissue engineering scaffold can also have an impact on how it interacts with the tissue into which it is placed. For example, nanofibers are able to closely mimic the morphology and structure of the ECM. This allows for better proliferation of cells through the scaffold and thus increasing the bioactivity of the system. For example, nanofibrous scaffolds have been formulated using poly (l-lactic acid)-co-poly (ɛ-caprolactone) and gelatin. The scaffolds were then plasma treated. Results showed that the plasma-treated scaffold was able to support the proliferation of fibroblasts as well as the secretion of collagen. This bioactive ability highlighted the suitability of nanofibrous scaffold for skin tissue engineering purposes [93]. 

## 7. Future Prospects

As has been discussed throughout this article, many studies are being performed in the field of tissue engineering. However, there is still more research that needs to be done, both within the general field and within the avenues of each specific tissue. For example, although many examples of innovative cartilage tissue regeneration systems have been mentions, unless these are further developed into accessible, simple and, most importantly, cost-effective systems, it is likely that surgeons will continue with the traditional treatment methods that are currently being used. In some cases, more extensive research is required in order for these systems to be on mechanically on par with native cartilaginous tissues [94].

Part of the further development of tissue regenerative systems is reliant on a deeper understanding of the processes by which these systems are formulated such as the exploration of in situ bioprinting. This would allow for scaffolds to be printed directly into the damaged site of a living model. Limited research has been done into this using inkjet-based bioprinting and laser-based bioprinting. It is believed that in using in situ bioprinting, native cells will be able to interact with the printed tissues and vice versa as it is being created for improved acceptance of the scaffold. This is an intriguing concept which could provide major benefits to tissue engineering science [95]. 

The progress that has been made in terms of in vitro tissue engineering in recent years has aided in the development of more novel biomaterial systems. The use of these components has allowed for the formulation of systems that are safer in terms of producing an immune response [96]. Researchers are able to enhance the full benefit of these materials in tissue engineering studies going forward.

Many of the studies which are mentioned in this article, though successful, are primarily proof of concept studies. There are some systems which have been tested in animal models but these need to be furthered in human testing and clinical trials. Animal model studies are able to confirm the way in which the tissue engineering systems reacts within an *in vivo*. The results of such studies provide researchers with a further insight into both the biocompatibility and biodegradability [97]. 

Once these further studies are completed successfully, the next challenge lies in how to evolve the working formula into a viable healthcare technology. The main complication in this is how to upscale the in many cases highly specific scientific process into a product which can be produced on a mass scale. Although some systems have made it to the market, it is currently a very small percentage of those which are being developed. There is much work that needs to be done in order for tissue engineering systems to meet the current demand [98]. 

## 8. Conclusions

There is much research being done into the field of tissue engineering. The variety of different systems and scaffold which are available to researchers have allowed them to be able to design systems which can be finetuned to suit the specific needs of the tissue type and site. This could include adequate mechanical strength for bone tissue engineering or flexibility for soft tissues.

The recent success of the tissue engineering developments has been made furthered even more by the incorporation of biopolymers. These systems are able to be formulated in such a way that they are biocompatible, which is a major influence on the success of the system. The cost effectiveness of biopolymers and the fact that they are readily available also make them ideal for use in these systems. They are able to be used in combination to highlight certain qualities that are favorable to the desired characteristics.

The recent successes of tissue engineering systems in furthered by the inclusion of stem cells. Although there are ethical considerations with the implication of stem cells, they provide promising results in terms of tissue regeneration. Further enhancing the research and utilization of stem cells is a promising step for the field of tissue engineering. 

One of the tissue systems which can especially benefit from the advances in tissue engineering technology is the eye. These new systems are particularly beneficial due to the fact that the eye is an immune privileged site which does not regenerate cells effectively. The developments being made are able to create an alternative to donor transplants which are very limited in numbers. 

Although more research is required, the work that is being done in both tissue regeneration and biopolymer science is promising and could have a positive impact on many lives around the world.

## Figures and Tables

**Table 1:** Outline of the properties to be considered when tissue engineering systems for regenerative medicine.

Properties	Design Considerations
Biocompatibility	The compatibility of a scaffold with the cells is of paramount importance. The scaffold should not illicit an immune response when inserted into the body.
Biodegradability	The ability of a scaffold to be biodegraded either through enzymatic or hydrolytic action is advantageous.
Electrical conductivity	Scaffolds which are conductive are able to influence the behavior of cells as a response to the electrical signals present in cell signaling.
Morphology	The morphology of the scaffold is vitality important as it impacts how the cells interact with the scaffold. The porosity of the scaffold ensures that cell infiltration can occur as well as the transfer of nutrients through the system.
Mechanical characteristics	This refers to characteristics such as the stiffness, elasticity and relaxation modulus of the scaffold. These influence cell behavior as well as the ability of the scaffold to mimic the natural microenvironment.
Ease of manufacturing	The cost of manufacturing, ease of the processes and the storage requirements are all factors which must be considered if the scaffold is to be produced on a large scale.

## Data Availability

Not applicable.
